# Increased adaptive potential in novel environments can be predicted from genetic variance in development time expressed in native environments

**DOI:** 10.1093/evlett/qrag012

**Published:** 2026-04-02

**Authors:** Greg M Walter, Keyne Monro, Alastair Wilson, Delia Terranova, Enrico la Spina, Maria Majorana, Giuseppe Pepe, Sarah du Plessis, James Clark, Salvatore Cozzolino, Antonia Cristaudo, Simon J Hiscock, Jon Bridle

**Affiliations:** School of Biological Sciences, University of Bristol, Bristol, United Kingdom; School of Biological Sciences, Monash University, Melbourne, Australia; School of Natural Sciences, University of Tasmania, Hobart, Australia; School of Biological Sciences, Monash University, Melbourne, Australia; Centre for Ecology and Conservation, University of Exeter, Cornwall, United Kingdom; Department of Biology, University of Naples Federico II, Naples, Italy; Department of Biological, Geological and Environmental Sciences, University of Catania, Catania, Italy; Department of Biological, Geological and Environmental Sciences, University of Catania, Catania, Italy; Department of Biological, Geological and Environmental Sciences, University of Catania, Catania, Italy; Department of Biological, Geological and Environmental Sciences, University of Catania, Catania, Italy; School of Biological Sciences, University of Bristol, Bristol, United Kingdom; Department of Plant Sciences, University of Oxford, Oxford, United Kingdom; Milner Centre for Evolution, University of Bath, Bath, United Kingdom; Department of Biology, University of Naples Federico II, Naples, Italy; Department of Biological, Geological and Environmental Sciences, University of Catania, Catania, Italy; Department of Plant Sciences, University of Oxford, Oxford, United Kingdom; School of Biological Sciences, University of Bristol, Bristol, United Kingdom; Department of Genetics, Evolution and Environment, University College London, London, United Kingdom

**Keywords:** additive genetic variance, early life history, genotype-by-environment interactions, novel environments, selection gradient, development costs

## Abstract

While developmental plasticity helps organisms to maintain fitness as environments change, such plasticity has limits. When novel environments exceed these limits and mean fitness declines, the extent of decline is expected to vary among genotypes, which could increase adaptive potential. We lack fundamental insights into whether genetic variation in early development is linked to adaptive potential in novel environments, which limits our ability to predict how natural populations will respond to global change. Using a breeding design, we generated c. 20,000 seeds of 2 ecologically contrasting Sicilian species of daisies (*Senecio*, Asteraceae) adapted to high and low elevations on Mount Etna. We planted the seeds across 4 elevations that included elevations within the native range of each species, the edge of their range, and a novel elevation. We tracked seedling mortality and measured development time as the number of days it took seedlings to establish. As predicted, genetic variance in survival increased at novel elevations, suggesting that adaptive potential consistently increases for contrasting species facing different novel environments. However, genetic variance in development time showed the opposite trend, decreasing at novel elevations. A strong negative genetic correlation between development time in the native range and survival at novel elevations suggested that genotypes with faster development in native environments survived better in novel environments. These results were consistent across the two ecologically contrasting species, suggesting that genetic variance in early development in native environments could be used to predict genotypes that increase adaptive potential in novel environments.

## Introduction

As global change creates increasingly novel environments, natural populations will need to shift their geographical ranges or adapt if they are to persist (Bridle & Hoffmann, 2022; Urban et al., 2024). Plasticity, the ability of genotypes to change their phenotype as the environment varies, provides an immediate response that can help populations to maintain fitness as environments change (Charmantier et al., 2008; Via, 1993; Via et al., 1995). However, plasticity is only adaptive when it helps genotypes to buffer familiar variation in the environment, which means that plasticity should become maladaptive in novel environments (Ghalambor et al., 2007). Theory predicts that when plasticity becomes maladaptive and population mean fitness declines, genotypes should vary in their sensitivity to the new environment, which increases genetic variation in fitness and therefore the adaptive potential of the population (Chevin et al., 2010; Hermisson & Wagner, 2004; Lande, 2009). We lack empirical tests of how genetic differences in fitness emerge when the limits to adaptive plasticity are exceeded, which remains a critical gap for predicting the adaptive capacity of populations facing global change.

Plasticity during early development can alter life history traits to match changes in the environment, which influences whether juveniles reach maturity and reproduce (Pålsson et al., 2024; Smallegange, 2022; Snell-Rood et al., 2018; Sultan, 2007). While plasticity in early development is widespread, it is unlikely to be adaptive in novel environments (Acasuso-Rivero et al., 2019; Ghalambor et al., 2007, 2015). Biophysical constraints can create developmental limits that determine the extent to which plasticity can change life history traits (Angert et al., 2008, 2014; Eriksson & Rafajlovic, 2022; Khare et al., 2024), and trade-offs among traits can limit the ability for plasticity to approach a phenotypic optimum for any given trait (DeWitt et al., 1998; Murren et al., 2015; Van Buskirk & Steiner, 2009). For example, while increases in temperature favor faster development, physiological limits determine the maximum rate of development, and large changes in development rate can incur trade-offs with survival, development or maintenance (Arendt, 1997; Willi & Van Buskirk, 2022). This means that although plasticity may allow genotypes to drastically shift their development in novel environments, such plasticity can incur fitness costs that reduce survival (Botero et al., 2015; Cotto et al., 2019; Schneider, 2022; Van Kleunen & Fischer, 2005; Walter et al., 2018). The extent to which these costs vary among genotypes is then expected to influence how genetic variation in fitness is expressed in novel environments, and whether adaptive potential increases (Ghalambor et al., 2007; Hoffmann & Sgrò, 2011; Nussey et al., 2005). However, we lack experiments that connect genetic variance in fitness with early life history traits in natural environments, especially as conditions reach, and then exceed, current ecological margins (Angert et al., 2014, 2020).

**Figure 1:**
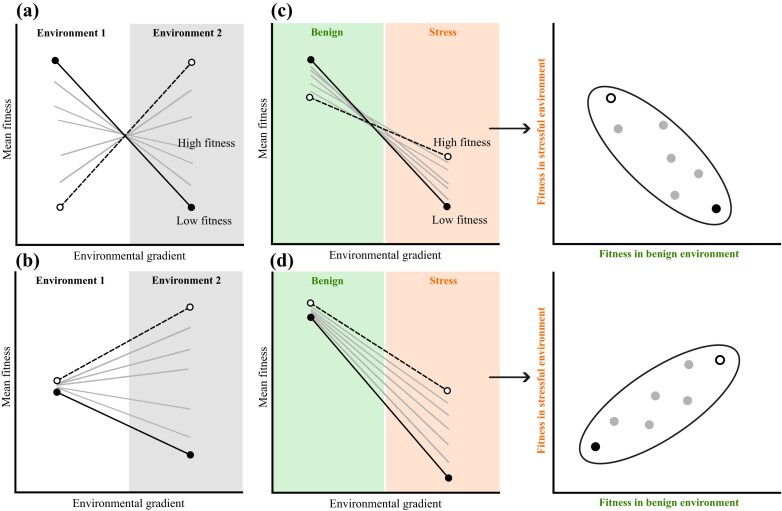
Genotype-by-environment interactions (G × E) for hypothetical genotypes that are represented by different lines, and with emphasis on the extreme differences in responses (dashed lines with open circles versus solid lines with closed circles). G × E can occur across environments as (a) changes in rank of genotypes across environments and/or (b) as changes in variance among genotypes across environments. (c and d) When faced with a stressful environment that reduces mean fitness, G × E means that some genotypes could suffer less under stress (dashed lines with open circles), while other genotypes suffer large reductions in fitness (solid lines with closed circles). This produces the same pattern of G × E (as in a and b), but includes reductions in mean fitness. Right-hand panels in c and d show the particular case where a change in genotype rank produces a negative genetic correlation across environments, whereas a change in variance produces a positive correlation. Note that it is possible for a combination of (c) and (d) to determine the expression of genetic variance in fitness in novel environments.

Genetic differences in plasticity emerge as genotype-by-environment interactions (G × E), which manifest as a change in rank order of the genotypes across environments (Figure 1a), and/or as a change in among-genotype variance across environments (Figure 1b; Mackay, 1981; Via & Lande, 1985). Although we know that patterns of G × E change depending on the trait and environmental context (Culumber et al., 2015; Merilä & Fry, 1998; Saltz et al., 2018; Sultan, 2007), we have a poor understanding of whether G × E differs for developmental traits compared to fitness-related traits, particularly under field conditions. Linking genetic variation in early life history with adaptive potential as environments become novel is therefore challenging, but crucial if we are to understand the adaptive capacity of populations and ecological communities that are being pushed beyond their current ecological margins (Anderson et al., 2012).

Genetic differences in fitness and developmental sensitivity to the environment can be created by a combination of both forms of G × E. Genetic correlations in fitness between native and novel environments can weaken or even become negative as environments become novel, in the latter case genotypes with high fitness in novel environments have lower fitness in native environments (Figure 1c; Angert et al., 2008; Walter et al., 2023). Novel environments themselves may also induce the expression of hidden genetic variation that increases genetic variance in fitness in novel environments (Figure 1d; Hermisson & Wagner, 2004; Paaby & Rockman, 2014; Shaw & Shaw, 2014; Walter et al., 2023). Fisher’s (1930) fundamental theorem of natural selection quantifies the potential for adaptation as the amount of additive genetic variance in relative fitness, ${V_A}( \omega )$, the ratio of genetic variance in absolute fitness to mean fitness. Evidence suggests that adaptive potential changes with the environment (Sheth et al., 2018), and that natural populations harbor genetic variance in fitness within their native range (Bonnet et al., 2022; Hendry et al., 2018; Kulbaba et al., 2019; Peschel et al., 2020). However, we have a limited understanding of how ${V_A}( \omega )$ changes across environmental gradients. Although some evidence suggests that ${V_A}( \omega )$—and therefore adaptive potential—can increase in novel or stressful environments (Torres-Martínez et al., 2019; Walter et al., 2023), meta-analyses remain equivocal (Charmantier & Garant, 2005; Hendry et al., 2018; Hoffmann & Merilä, 1999). We therefore require field studies that use species adapted to contrasting habitats with different life history strategies to test how genetic variance in early life history determines fitness across an ecological gradient that includes native and novel elevations.

Although theory suggests that cryptic genetic variation should emerge in novel environments, causing G × E to increase genetic variance in fitness (McGuigan & Sgrò, 2009; Paaby & Rockman, 2014), predicting how G × E emerges at early life history stages is more difficult. Strong selection during early development often removes unfit genotypes before they can be measured, which makes quantifying genetic variance in early life history traits challenging (Hadfield, 2008; Kruuk et al., 2008; Wadgymar et al., 2017). So while we know that evolutionary potential in early life history traits can change with the environment (Chantepie et al., 2024), we still do not know whether genetic variance in early life history traits increases in novel environments (Charmantier & Garant, 2005; Garant et al., 2008; Riley et al., 2023; Wood & Brodie, 2015). Intuitively, we would predict genotypic variation in early development to translate into variation in fitness, and so we would expect increased genetic variance in fitness in novel environments to be correlated with a similar increase in genetic variance in early development traits. To predict the response of natural populations facing global change, we therefore need to understand how G × E in early life history traits changes across environments, and whether such changes determine adaptive potential in novel environments (Chirgwin et al., 2021).

To test how genetic variance in fitness and early development change as environments become novel, we generated seeds of two closely related species of Sicilian daisy (*Senecio*, Asteraceae), which we planted along an elevational gradient on Mt. Etna. *Senecio chrysanthemifolius* is a short-lived perennial with dissected (more complex) leaves that occupies disturbed habitats (e.g., roadsides) at 400–1,500 m.a.s.l (meters above sea level) on Mt. Etna. By contrast, *Senecio aethnensis* is a longer-lived perennial with entire glaucous (simpler and waxy) leaves endemic to lava flows above 2,000 m.a.s.l on Mt. Etna, where individuals grow back each spring after being covered by snow in winter (Walter et al., 2020). Species also differ in plasticity in leaf traits, with *S. chrysanthemifolius* exhibiting a greater capacity for adaptive plasticity when faced with novel environments compared to *S. aethnensis* (Walter et al., 2022, 2024). Here, we use data derived from a seed transplant experiment that tested how genetic variance in multivariate leaf phenotypes changed across elevations (Walter et al., 2024). In this previous study, we found that changes in genetic variance were associated with low adaptive potential for the high-elevation *S. aethnensis* seedlings planted at low elevations, whereas the low-elevation *S. chrysanthemifolius* displayed smaller changes in genetic variance and a greater adaptive potential at the novel high elevation.

We used a quantitative genetic breeding design to produce seeds for c. 100 families of each species, which we reciprocally planted across an elevational gradient that included the native elevation of each species and two intermediate elevations. For seedlings that emerged, we tracked mortality, and measured development time as the time it took seedlings to establish (produce 10 leaves). With these data, we first tested for patterns of adaptive divergence in survival, emergence, and seedling establishment between the native habitats. We then tested three hypotheses to understand how genotypes of each species varied in development time and survival across the ecological gradient. *Hypothesis I:* If development time is important for maintaining survival across elevations, we expected selection on development time to change across elevations similarly for both species. *Hypothesis II:* As environments become novel and plasticity can no longer maintain fitness, we predicted that declines in survival would be associated with stronger patterns of G × E in both development time and survival. We therefore expected that as environments became novel, genetic variance would increase, and genetic correlations would change from strongly positive (+1) between environments within the native range, to weakly positive (e.g., 0.2–0.5) or negative between native and novel environments. *Hypothesis III:* If patterns of G × E in development time are linked to survival, we would detect strong genetic correlations between development time and survival at novel elevations.

## Methods and materials

### Breeding design and field experiment

To produce seeds of both species, we first collected cuttings from c. 80 mature individuals from natural populations of both species. For *S. chrysanthemifolius*, we sampled individuals from five locations at the base of Mt. Etna (Table S1 and Figure S1). For *S. aethnensis*, we sampled individuals from a range of elevations on both the North and South of Mt. Etna in October 2018 (Table S1 and Figure S1). We chose this sampling design because individuals of each species either occur in small numbers in patches of habitat that are close together (*S. chrysanthemifolius*; Walter et al., 2023) or occur at low density across their elevational range (*S. aethnensis*), and so we aimed to obtain enough individuals for the breeding design while reducing the chances of sampling closely related individuals. All sampling locations within each species were in close proximity (largest distances between sites: c. 6 km for *S. chrysanthemifolius*, and c. 10 km for *S. aethnensis*; Figure S1), and because both species are pollinated by wide-ranging insects (particularly hoverflies) and possess wind-dispersed seeds, gene flow should be high. Our samples therefore represent collections from a single population within each species rather than distinct subpopulations, which minimizes the potential for heterosis or outbreeding depression to influence crosses within each species.

**Figure 2:**
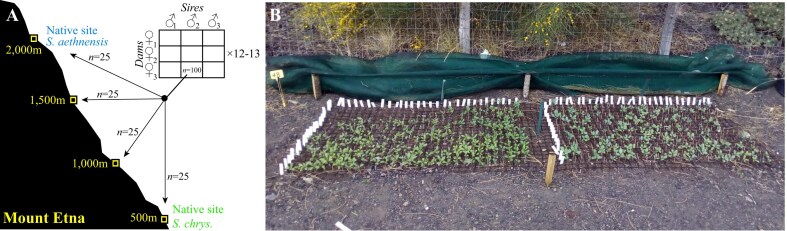
The experimental design, reproduced from Walter et al. (2024). (a) For two Etnean *Senecio* species, we mated 36–38 sires each to three dams, and produced 100 seeds from each mating (family). We planted 25 seeds from each family at each of four elevations that represent the native site of each species and two intermediate elevations, and tracked the emergence of each seedling, development time to establishment, and survival. (b) Photo of an experimental block at 2,000 m with seedlings of both species 8 weeks after sowing (*S. chrysanthemifolius* on left).

We propagated one cutting from each field-collected individual in the glasshouse. When plants produced buds, we covered branches with perforated bread bags to exclude pollinators while providing airflow. For each species, we randomly assigned half of the individuals (which are hermaphroditic and self-incompatible) as females (dams), the other half as males (sires). We then used a factorial design to randomly mate sires to dams in 3 × 3 blocks (three sires mated to three dams per block; Figure 2a) to produce 94 families of *S. aethnensis* (*n* = 36 sires, *n =* 35 dams) and 108 families of *S. chrysanthemifolius* (*n =* 38 sires, *n* = 38 dams). We performed crosses by removing flowerheads from sires and rubbing their anthers on flowerheads of dams. Fertilized flowerheads were labelled and covered with mesh bags to catch seeds. Due to limitations in glasshouse space, we performed crosses on each species separately: *S. chrysanthemifolius* in January to February 2018, and *S. aethnensis* in January to March 2019.

In April 2019, we planted 25 seeds per family at each of four elevations on Mt. Etna that included the native habitats of both species (500 and 2,000 m) and two intermediate elevations (1,000 and 1,500 m) (Figure 2b; Walter et al., 2022, 2024). Vagrant individuals of these species are often found between 1,000 and 1,500 m, which means these intermediate elevations could support range expansions. At each elevation, we randomized seeds of each family into five experimental blocks (*S. aethnensis n* = 432 seeds/block, *n =* 2,160 seeds/site; *S. chrysanthemifolius n =* 540 seeds/block, *n =* 2,700 seeds/site; total *N* = 19,232 seeds). However, *S. aethnensis* grew slowly and produced too few flowers in the glasshouse to obtain 100 seeds for each family, meaning that 4, 8, and 10 families were represented by 10, 15, and 20 seeds per elevation, respectively.

To prepare each experimental block, we cleared the ground of plant matter and debris, and placed a plastic grid (4 cm^2^ cells) on the ground. We attached each individual seed to the middle of a toothpick using nondrip superglue and then pushed each toothpick into the soil of a single grid cell so that the seed sat 1–2 mm below the soil surface (Walter et al., 2016). To replicate natural germination conditions, we suspended 90% shade cloth 20cm above each plot and kept the seeds moist until seedling emergence ceased (2–3 weeks). After this initial period, we replaced the shade cloth with a lighter 40% shade cloth to replicate shade that naturally growing plants are often found under. This approach maintained a strong environmental effect at each elevation without exposing plants to extreme environmental conditions. It also allowed us to initiate the experiment synchronously across all sites and genotypes while simulating the high-moisture, shaded conditions that typically trigger germination following rainfall events in natural populations.

For the seedlings that emerged, we focused on recording mortality and the time it took seedlings to establish (produce 10 leaves). The experiment concluded in January 2020 when mortality stabilized at all sites (Figure S2) and plants started growing into each other, which increased competition and would have biased further data collection.

### Statistical analyses

All analyses below were performed in R version 4.4.1 (R Core Team, 2024). Throughout the analyses, we treated elevation as categorical (rather than continuous) so that we could test how development time and survival change across elevation as environments moved from native to novel. We first assessed how the *Senecio* species differed in emergence, survival, and development across elevations, and then tested the three hypotheses described in the *Introduction*.

### Quantifying mean differences in seedling traits across elevations

To test how both species emerged, survived, and developed across the elevation gradient, we used *glmmTMB* (Brooks et al., 2017) to apply the linear mixed model


(1) \begin{eqnarray*}
{y_{\mathit{ijklm}}} = S{p_i} \times {E_j} + {b_k} + {f_l} + {e_{m\left( {\mathit{ijkl}} \right)}},
\end{eqnarray*}

where the fixed effects include $S{p_i}$ × ${E_j}$ as the *i*th species (*Sp*) planted at the *j*th elevation (*E*) included as categorical variables. Random effects include ${b_k}$ as the *k*th experimental block, ${f_l}$ as the *l*th family within species, and ${e_{m( {ijkl} )}}\,\,$ represents the residual. We used type III ANOVA to test for significant elevation × species interactions, and *emmeans* (Lenth, 2019) to obtain marginal means for each species at each elevation.

We applied Equation 1 separately for emergence, seedling establishment, and survival to the end of summer, with each variable included as a binary response variable (${y_{\mathit{ijklm}}}$) using a logit link function. For each trait, Equation 1 estimated the species mean at each elevation. Seedling establishment and survival excluded seeds that failed to germinate and emerge, and so only represent fitness of the seedlings that successfully emerged. However, preliminary analyses suggested that including variation in emergence success did not change our results or their interpretation. To understand how development time changed across elevations, we applied Equation 1 with development time as a Gaussian-distributed response variable. To check whether we obtained the same results while accounting for dependencies across life history stages, we modeled emergence and survival together using *Aster* (Geyer et al., 2007, 2013).

### Hypothesis I: selection on development time changes across elevations

To quantify selection on development time for each species and elevation, we used *glmmTMB* to apply the generalized linear mixed model


(2) \begin{eqnarray*}
{y_{\mathit{ijklm}}} = \mathit{Development}\,\,\mathit{time} \times S{p_i} \times {E_j} + {b_k} + {f_l} + {e_{m\left( {\mathit{ijkl}} \right)}},
\end{eqnarray*}

where fixed effects include the interaction between development time, species, and elevation. We included survival as the binomially distributed response variable (${y_{\mathit{ijklm}}}$) with a probit link function. For the seedlings that successfully established, this estimates directional selection on development time based on differences in survival. Random effects include ${b_k}$ as the *k*th block and ${f_l}$ as the *l*th family within species, and ${e_{m( {\mathit{ijkl}} )}}$ is the residual. We then transformed the coefficients from the link to the data scale and divided them by the mean survival so that they represent selection gradients (Janzen & Stern, 1998). A significant three-way interaction ($Development\,\,\mathit{time} \times \mathit{Elevation} \times S{p_i}$) would suggest that selection on development time differs between species and changes across elevation.

### Hypothesis II: G × E in survival and development time increases in novel environments

To estimate additive genetic variance in both survival and development time, we used *MCMCglmm* (Hadfield, 2010) to apply


(3) \begin{eqnarray*}
{y_{\mathit{ijklm}}} = {T_i} + {s_j} + {d_{k\left( j \right)}} + {b_{l\left( i \right)}} + {e_{m\left( {\mathit{ijkl}} \right)}},
\end{eqnarray*}

where transplant elevation (${T_i}$) was the only fixed effect. Random effects included ${s_j}$ as the *j*th sire, ${d_{k( j )}}$ the *k*th dam nested within sire, ${b_{l( i )}}$ the variance among blocks within a transplant elevation, and ${e_{m( {\mathit{ijkl}} )}}$ the residual variation. We use a nested approach to estimate additive genetic variance as it reduces the number of parameters estimated by omitting the sire × dam interaction, which becomes incorporated into the residual variance without inflating the additive genetic variance (Schielzeth & Nakagawa, 2013; Walter et al., 2024). We then calculated additive genetic variance as four times the sire variance (Lynch & Walsh, 1998). By including transplant elevation as a fixed effect and specifying unstructured covariance matrices for the sire component, we estimated a 4 × 4 matrix with variance at each elevation along the diagonal and covariances among elevations on the off-diagonals. To estimate the genetic correlations between elevations, we transformed the covariances into correlations using the *cov2cor* function.

We used Equation 3 to estimate genetic variance in survival and development time separately. Response variables (${y_{\mathit{ijklm}}}$) therefore included development time (Gaussian) and survival (binary) as the presence/absence of plants after mortality stabilized at the end of summer (28 August; Figure S2). For survival we specified a binomial error distribution with a probit link function. We used chains with a burn-in of 200,000 iterations, a thinning interval of 2,000 iterations and saving 1,000 iterations that provided the posterior distribution for all parameters estimated. We confirmed model convergence by checking the chains mixed sufficiently well, that autocorrelation was lower than 0.05, and that our parameter-expanded prior was uninformative (Hadfield, 2010). Given that *MCMCglmm* constrains variances to be positive, we tested whether our estimates of genetic variance were statistically significant by comparing our observed estimates to a null distribution, which we created by randomly shuffling the survival and development time data among the families 200 times and re-estimating genetic variance for each randomized dataset (Walter et al., 2024). Where our observed estimates exceeded the null distribution provides evidence that we captured statistically significant genetic variation for a trait estimated in a given environment.

To quantify changes in genetic variance in survival across elevations, we first back-transformed estimates of variance from the link scale to the data scale using *QGlmm* (de Villemereuil et al., 2016). For both traits, we then calculated genetic variance in relative survival and development time at each elevation by dividing absolute variance for each trait by the squared mean estimate of each trait. This represents genetic variance relative to the mean at each transplant elevation, and for survival this represents adaptive potential (Hansen et al., 2011; Houle, 1992). We checked the consistency of these results using random-effects models with *Aster*.

### Hypothesis III: G × E in development time is associated with survival in novel environments

To quantify genetic correlations between development time and survival, we used *MCMCglmm* to apply Equation 3, but using the data collected within transplant elevation (and so removed the fixed effect of transplant elevation). Instead, we applied a bivariate model that included both development time and survival as response variables. By specifying an unstructured covariance matrix for the sire component, we estimated (for each species) a 2 × 2 matrix representing genetic variance in each trait and the genetic covariance between the traits. We then converted the covariances to correlations to quantify the strength of genetic correlations between development time and survival.

## Results

### Evidence of adaptive divergence between species from elevational extremes

**Figure 3:**
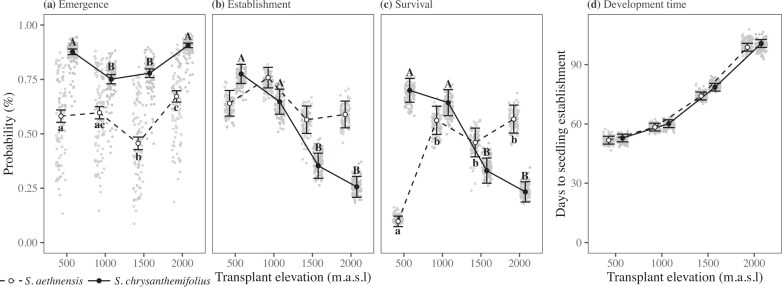
Changes in seedling traits for the two Etnean *Senecio* species planted across an elevation gradient. Lowercase and uppercase letters denote significant differences (α = 0.05 with Bonferroni adjustment) across elevations for *S. aethnensis* (open circles and dashed lines) and *S. chrysanthemifolius* (closed circles and solid lines), respectively. (a) Seedling emergence was greater for *S. chrysanthemifolius*, which was greater at the elevational extremes. Emergence was greatest for *S. aethnensis* at its native elevation (2,000 m). (b) The probability of seedling establishment remained similar across elevations for *S. aethnensis*, but was reduced at higher elevations for *S. chrysanthemifolius*. (c) As evidence of adaptive divergence, both species survived to the end of summer (28 August) better at their home site than the foreign species, and better at their home site than at the novel elevation. (d) Development time was slower at higher elevations (all elevations were significantly different from each other) with *S. aethnensis* showing slightly faster development than *S. chrysanthemifolius*.

Across all elevations, average seedling emergence was 75%–85% for *S. chrysanthemifolius* and 50%–70% for *S. aethnensis* (Figure 3a). For *S. aethnensis*, c. 60% of seedlings established at all elevations, which contrasted with *S. chrysanthemifolius*, where 75% of seedlings established at the home site but gradually reduced across elevations to 25% at the 2,000-m elevation (Figure 3b). Mortality stabilized after summer (Figure S2), at which point evidence of adaptive divergence between the two species emerged. Both species showed greater probability of survival within their native range compared to a novel elevation, which is the native habitat of the other species (species × elevation *χ^2^*(3) = 978.71, *p* < 0.001; Figure 3c). At each of the native habitats (500 and 2,000 m), the native species survived better than the species from the other habitat (Figure 3c). Therefore, *S. chrysanthemifolius* survived better than *S. aethnensis* at low elevations (500 m *Z* = 18.7, *p* < 0.001; 1,000 m *Z* = 3.2, *p* = 0.0014), and *S. aethnensis* survived significantly better at higher elevations (1,500 m *Z* = 8.4, *p* < 0.001; 2,000 m *Z* = 14.9, *p* < 0.001). Modeling emergence and survival together using *Aster* recovered similar patterns of adaptive divergence between the native habitats of both species (Table S2).

### Hypothesis I: selection on development time changes across elevations similarly for both species

Development time changed significantly across elevations (*χ^2^*(3) = 357.39, *p* < 0.001), with seedlings establishing faster at lower elevations (Figure 3d). A significant interaction between development time and elevation suggested that species responded differently to elevation (*χ^2^*(3) = 9.06, *p* = 0.0285). Seedlings of *S. aethnensis* tended to establish earlier than *S. chrysanthemifolius*, particularly at higher elevations (Figure 3d).

Estimating selection on development time, we found no evidence of a significant three-way interaction between development time, species, and elevation (*χ^2^*(3) = 2.59, *p* = 0.459). However, we observed a significant development time × elevation interaction (*χ^2^*(3) = 47.33, *p* < 0.001), suggesting that selection on development time changed across elevations but did so similarly for both species. Selection favored faster development at 500 m, but slower development at 2,000 m (Table 1). Selection on development time in the novel environments was therefore in the direction of plasticity for *S. chrysanthemifolius* at 2,000 m (slower development at higher elevations), as well as for *S. aethnensis* at 500 m (faster development at lower elevations).

**Table 1.** Selection on development time across elevations for both species.

Elevation	Species	$\beta $	Development time	*SE*	95% confidence interval
500 m	*S. aethnensis*	**−0.030**	**−0.33**	**0.13**	**−0.59, −0.07**
	*S. chrysanthemifolius*	**−0.043**	**−0.48**	**0.1**	**−0.68, −0.29**
1,000 m	*S. aethnensis*	0.007	0.08	0.09	**−**0.1, 0.26
	*S. chrysanthemifolius*	−0.016	−0.17	0.24	−0.65, 0.3
1,500 m	*S. aethnensis*	0.004	0.05	0.13	−0.2, 0.29
	*S. chrysanthemifolius*	−0.004	−0.05	0.18	−0.41, 0.31
2,000 m	*S. aethnensis*	**0.118**	**1.31**	**0.31**	**0.71, 1.92**
	*S. chrysanthemifolius*	**0.053**	**0.59**	**0.18**	**0.24, 0.94**

*Note. β* represents the standardized selection gradient. Significant estimates of selection are denoted in bold (*p* < 0.05). Selection was only significant at elevational extremes, and in the same direction for both species.

### Hypothesis II: G × E across elevation differed for development time and survival

**Figure 4:**
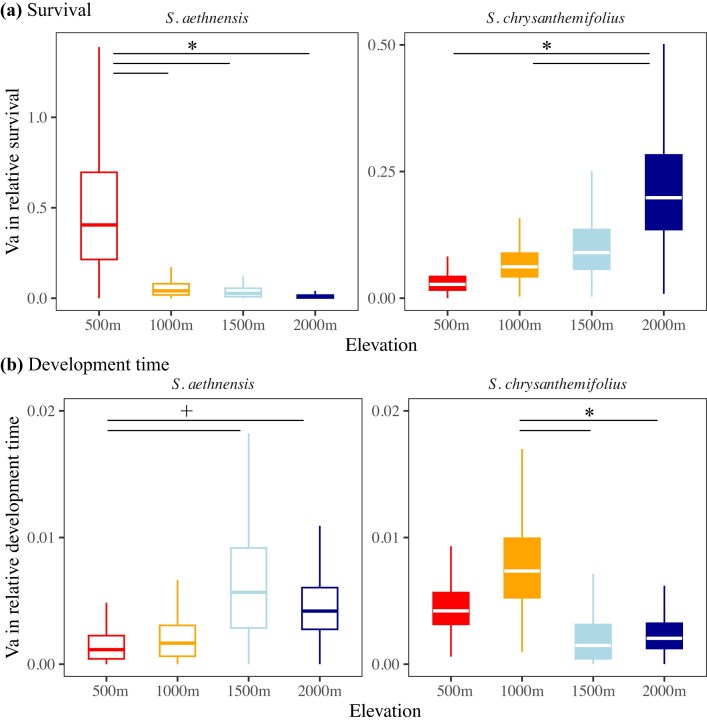
Genetic variance in (a) survival and (b) development time to seedling establishment for both species at all elevations. (a) At their native elevations, genetic variance in survival was near zero, but greater at novel elevations. (b) For both species, genetic variance in development time was lower at novel elevations compared to native elevations. Horizontal lines with asterisks denote elevations showing strong evidence of differences in genetic variance, where there is >90% posterior probability that one variance exceeds the other. Plus (+) signs show distributions where there is >85% posterior probability that one variance exceeds the other.

We predicted that stronger patterns of G × E would emerge at elevations further from the home site of each species. We therefore expected genetic variance to increase and genetic correlations between the home site and other elevations to weaken as environments became novel. We observed the predicted pattern for survival. Genetic variance in survival was near zero at the native elevations of both species and significantly greater at the novel environments (Figure 4a). Genetic variance increased consistently with elevation for *S. chrysanthemifolius*, but remained close to zero across most elevations for *S. aethensis* and only increased at 500 m (where mean survival was lowest; Figure 3c). These results were consistent when we used *Aster* to account for dependencies across life history traits (Table S2). For development time, we found the opposite pattern to our original prediction. For both species, genetic variance in development time was more than double at elevations within the native range compared to near-zero genetic variance at the range edge and the novel environment (Figure 4b). While this change in genetic variance in development time across elevation was significant for *S. chrysanthemifolius*, it was only marginally significant for *S. aethnensis* (Figure 4b). However, our observed estimates of genetic variance in survival (in novel environments) and development time (in native environments) exceeded the null distribution for both species and traits (Figure S3), suggesting biologically meaningful increases in genetic variance within the native range for development time, and in the novel environment for survival.

Genetic correlations in survival across elevations were all >0.7 for *S. chrysanthemifolius*, and ranged from 0.06 to 0.58 for *S. aethnensis* (Table S3). By contrast, genetic correlations for development time across environments ranged between 0.35 and 0.79 for both species (Table S3). This suggests that rank changes in genotype survival across elevations were likely to have only contributed moderately to G × E in this experiment, and mainly for survival in *S. aethnensis*. However, confidence intervals for the estimates of cross-elevation genetic correlations were often large and overlapping zero, particularly for survival in *S. aethnensis* (Table S3), and so care must be taken in their interpretation.

### Hypothesis III: genetic variation for faster development was associated with higher fitness in novel environments

**Figure 5:**
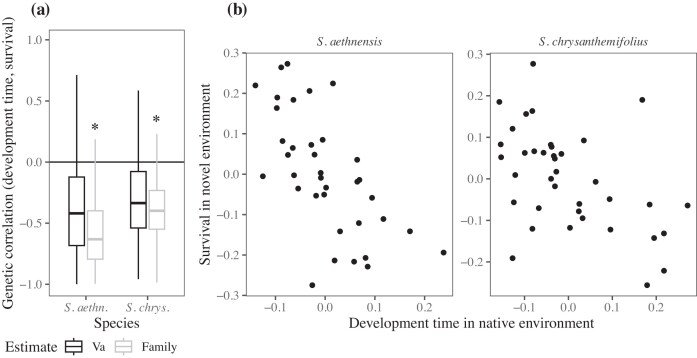
The genetic correlation between development time at a site in the native range and survival in the novel environment. (a) Boxplots represent the posterior distribution for the estimates of the genetic correlation, with asterisks denoting correlations where <10% of their distributions overlap. Due to the difficulty of estimating the cross-elevation genetic correlation, we included estimates for the among-family variance, which shows the same pattern but is estimated more precisely due to higher replication of families than sires. (b) Mean estimates for the sires (i.e., best linear unbiased predictors) that represent the genetic correlation across elevations. For both species, genotypes with faster development in the native range showed higher survival in the novel environment.

In our original hypothesis, we predicted that greater G × E in both development time and survival would emerge in novel environments. However, given that development time showed the opposite pattern (a reduction in genetic variance in novel environments), we tested whether genetic variance in development time within the native range was associated with genetic variance in survival at the novel elevations. This was necessary because estimating correlations where genetic variance is near-zero is uninformative. We found that both species showed strong negative genetic correlations between development time within the native range and survival at the novel elevation (Figure 5a). This suggests that genotypes that develop faster in native environments are associated with higher fitness in novel environments (Figure 5b).

## Discussion

We reciprocally planted seeds of two ecologically contrasting species of *Senecio* wildflowers across an elevational gradient. By tracking seedling survival and time to establishment, we found support for three hypotheses that reveal how adaptive potential changes from native to novel environments. First, selection on development time was environment-dependent and consistent for both species – selection favored faster development at 500 m but slower development at 2,000 m (Table 1). Second, both species showed high survival but low genetic variance in survival in their native elevations, suggesting that all genotypes could maintain similarly high survival within their native range (Figure 4a). However, where mean survival was reduced in novel environments, genetic variance in survival increased, which suggests a greater potential for adaptation to the novel environment (Figure 4a). Surprisingly, genetic variance in development time showed the opposite trend, with greater genetic variance observed within the native range compared to near-zero variance in novel environments (Figure 4b). Third, for both species, we detected negative genetic correlations between development time within the native range and survival in novel environments (Figure 5). Genotypes with faster development times in the native range therefore had greater survival in novel environments, which suggests that genetic variation in early life history traits can predict which genotypes could increase adaptive potential to novel environments. Our results therefore suggest that it could be possible to predict genotypes that could increase the resilience of populations to environmental change, which could benefit conservation strategies, as discussed below.

### Life history trade-offs and the increase in adaptive potential in novel environments

Comparing the response of two species with contrasting life history strategies revealed strikingly similar responses to different (low versus high elevation) novel environments. At each novel elevation, plasticity allowed both species to match the development time of the native species (slower development at higher elevations), but our results suggest that this exceeded the limits of plasticity, which created trade-offs with survival. Such fitness costs of plasticity are notoriously difficult to detect because selection may remove genotypes early in life, and because novel environments could produce a canalized stress responses that induces the same phenotype in all individuals (Auld et al., 2010; DeWitt et al., 1998; Murren et al., 2015). In particular, selection is expected to remove genetic variation in life history traits, which could have created the low levels of genetic variation in development time observed in our experiment (Angert et al., 2014). However, we provide an alternative explanation: increased genetic variance in fitness in novel environments could be created by genetic variation in the costs of plasticity itself. Specifically, genotypes that develop more quickly in their native range appear to incur lower developmental costs that lead to better survival in novel environments. This pattern held even in high-elevation environments that otherwise favored slower development, suggesting that the costs of extending early development under stressful or unpredictable conditions can outweigh the benefits of phenological matching (Walter et al., 2018).

Together, these results suggest that genotypes that increase the capacity to maintain fitness while expressing plastic responses may be an important but underappreciated source of adaptive potential in novel environments. While our experimental design used drastic environmental shifts to maximize detection of these effects, the underlying mechanism—where genotypes that develop efficiently under native conditions maintain higher fitness when conditions change—could also operate under more gradual environmental fluctuations common in nature. For annual plants, genotypes with plasticity patterns particularly suited to novel conditions could be produced each flowering season. For perennial plants (or animals), genotypes that develop more rapidly or efficiently in their native environments may be better able to withstand rapid environmental change. However, this interpretation depends on the life history of the organism. In contrast to our results, Ensing and Eckert (2019) found that high-elevation plants had faster life cycles to cope with more narrow growth windows compared to low-elevation species. Understanding how G × E determines adaptive potential for organisms that vary in life history strategies remains a goal for evolutionary ecology, particularly as we seek to predict which populations and species are most vulnerable to rapid environmental change.

### Evidence for increased adaptive potential in novel environments

Although stressful environments are expected to release cryptic genetic variation in phenotypes, evidence is rare (McGuigan & Sgrò, 2009; Paaby & Rockman, 2014). Meta-analyses have found little support for increased genetic variance in novel environments for morphological and developmental traits (Charmantier & Garant, 2005; Noble et al., 2019; Riley et al., 2023), but this could reflect inconsistencies in defining stress versus novelty, or variation across studies in the extent of the severity of stressful environments or treatments (Riley et al., 2023). We found contrasting patterns in the expression of genetic variance across traits: genetic variance decreased for development time but increased for survival in novel environments. Given survival is a direct component of fitness while development time affects fitness more indirectly through its effects on reproductive timing and competitive ability, our results suggest that environmental novelty may more readily release genetic variance in traits with direct fitness consequences. This pattern could arise if adaptive plasticity in development time within native environments breaks down in novel conditions, increasing genetic differences in stress tolerance and therefore survival. Whether these patterns remain if we consider other direct versus indirect components of fitness remains to be tested.

Both *Senecio* species showed a significant increase in genetic variance in fitness in novel environments. Although this is consistent with recent studies (Kulbaba et al., 2019; Torres-Martínez et al., 2019; Walter et al., 2023), and suggests that increased adaptive potential in novel environments could be a general pattern for natural populations, further work is needed to confirm this across species with varying levels of genetic diversity and habitat heterogeneity. We also have a poor understanding of the capacity for these genetic differences in relative fitness in novel environments to allow adaptation or persistence in response to environmental change. This uncertainty stems from the critical knowledge gap that we do not know the conditions (e.g., the level of stress and key environmental variables) that increase genetic variance in fitness, nor whether populations can persist and then adapt to those same conditions (Chevin & Bridle, 2025). Future research should therefore prioritize quantifying the stress thresholds necessary to induce genetic variance in fitness and determine whether mean fitness remains high enough under those conditions to prevent extinction. Such information would allow us to predict not only the potential for adaptation, but also population persistence, providing more accurate assessments of population resilience under rapid environmental change.

### Implications for predicting population and species’ persistence under environmental change

By connecting variation in development within the native range to survival in novel environments, we show that the genetic variation observed within the native range could be used to predict adaptive potential and the fitness responses of genotypes to novel environments. This is encouraging for conservation efforts because it means that it may be possible to identify genotypes that could help populations better cope with environmental change. However, further work needs to quantify how different early life history traits relate to lifetime fitness in novel environments, and how consistent this is across taxa and across different environmental stresses. Future research should target key traits and life history stages within native environments, and test whether they could be used to identify genotypes that will be more resilient to environmental change (Bay et al., 2017). Genomic prediction could then be used to identify adaptive genetic variation for novel environments created by climate change (Ashraf et al., 2022; Waldvogel et al., 2020). Such an approach can increase our ability to predict the adaptive capacity of populations facing environmental change as well as enhance conservation efforts seeking to identify genotypes that could aid population persistence. Furthermore, incorporating estimates of genetic diversity can test how demographic history determines the expression of genetic variance in novel environments, and in particular, identify whether populations with low genetic diversity simply lack the genotypes necessary to aid population persistence in novel environments, and whether such genotypes can then be acquired from elsewhere in the geographical range (Olazcuaga et al., 2023; Sgrò et al., 2011). Such information will be crucial for developing techniques for genetic rescue of threatened populations or species.

### Sources of G × E in development time and survival

Our results suggest that the emergence of cryptic genetic variation underlies increased adaptive potential in novel environments that we observed (McGuigan & Sgrò, 2009; Paaby & Rockman, 2014). However, we still do not know how epigenetic or gene regulation differences among genotypes underlie increased adaptive potential in novel environments (Liu et al., 2022; McGuigan et al., 2021). It is possible that increased genetic variance in fitness could be linked to conditionally neutral alleles that have zero fitness effects in native environments, but strong fitness effects in a novel environment because they have not been exposed to selection that would have otherwise removed them (Paaby & Rockman, 2014). Further experiments are therefore needed to identify the genetic and environmental mechanisms that increase adaptive potential in stressful or novel environments.

### Limitations of inferring adaptive potential using seed experiments

We provide strong evidence in two *Senecio* species that moving into novel environments where plasticity can no longer maintain fitness, genetic variance in fitness increases. This is consistent with our previous study that showed greater genetic variance in fitness measured as reproductive (flower) investment for *S. chrysanthemifolius* at the novel 2,000 m elevation Walter et al. 2023. Although tracking seedlings allows key aspects of early life traits to be included in our current study, we were unable to measure performance at later life history stages to capture traits related to lifetime fitness. This requires tracking plants from seeds to maturity and fecundity (e.g., Kulbaba et al., 2019; Peschel et al., 2020; Shaw et al., 2008; Sheth et al., 2018), which is a challenge for field experiments, but vital for understanding the potential for adaptation at range margins and in response to environmental change (Bridle & Hoffmann, 2022). Our genetic correlations between development time and survival were not significant for sire variance (but were for among-family variance), suggesting that further replication is needed to more precisely estimate additive genetic correlations across environments, particularly for different traits. Our estimates of genetic variance in development time also excludes seedlings that died early or failed to germinate, and so selection could have removed genetic variation that may have led to greater genetic variance in development time in novel environments, as originally predicted. However, this should only be an issue for *S. chrysanthemifolius* because selection on *S. aethnensis* at low elevations largely occurred after seedling establishment, meaning that development time was measured for most of the emergent seedlings. Future experiments should focus on estimating developmental and morphological traits in seedlings well before establishment (i.e., before selection removes variation) to better quantify how plasticity and adaptive potential could contribute to population persistence in novel environments.

### Conclusions

Using an extensive reciprocal planting of two contrasting *Senecio* wildflower species across an elevation gradient, we show that increased genetic variance in fitness in novel environments increases adaptive potential. We also show that G × E in early development showed the opposite trend, with greater genetic variance in seedling development time observed in native compared to novel environments. A strong negative genetic correlation between development time within the native range with fitness outside the range suggests that genotypes that develop faster within the range show greater fitness in novel environments. While further work needs to understand the mechanisms underlying this pattern, these data suggest that genetic variance in life history traits could predict genotypes that could aid population persistence in novel environments. This information should be useful for efforts to predict population and community persistence under rapid environmental change, and to identify genotypes that could help persistence and adaptation to novel environments created by global change.

## Supplementary Material

qrag012_Supplemental_File

## Data Availability

Data are available at https://doi.org/10.5061/dryad.k6djh9wdf. Codes to run all analyses are located at https://osf.io/m5u9f.
